# Analgesic and anti-inflammatory drug use and risk of bladder cancer: a population based case control study

**DOI:** 10.1186/1471-2490-7-13

**Published:** 2007-08-10

**Authors:** Joan Fortuny, Manolis Kogevinas, Michael S Zens, Alan Schned, Angeline S Andrew, John Heaney, Karl T Kelsey, Margaret R Karagas

**Affiliations:** 1 Respiratory and Environmental Health Research Unit. Municipal Institute of Medical Research (IMIM), 08003 Barcelona, Catalonia, Spain; 2 Department of Community and Family Medicine, Section of Biostatistics and Epidemiology, Dartmouth Medical School, Hanover, NH 03756, USA; 3 Department of Pathology, Dartmouth Medical School, Hanover, NH 03756, USA; 4 Department of Surgery, Dartmouth Medical School, Hanover, NH 03756, USA; 5 Departments of Community Health and Laboratory Medicine and Pathology, Brown University, PRovidence, RI 02912, USA

## Abstract

**Background:**

Use of phenacetin and other analgesic and non-steroidal anti-inflammatory drugs (NSAIDs) potentially influences bladder cancer incidence, but epidemiologic evidence is limited.

**Methods:**

We analyzed data from 376 incident bladder cancer cases and 463 controls from a population-based case-control study in New Hampshire on whom regular use of analgesic drugs and NSAIDs was obtained. Odds ratios and 95% confidence intervals were computed using logistic regression with adjustment for potentially confounding factors. Separate models by tumor stage, grade and TP53 status were conducted.

**Results:**

We found an elevated odds ratio (OR) associated with reported use of phenacetin-containing medications, especially with longer duration of use (OR _>8 years _= 3.00, 95% confidence interval (CI) = 1.4–6.5). In contrast, use of paracetamol did not relate overall to risk of bladder cancer. We also found that regular use of any NSAID was associated with a statistically significant decrease in bladder cancer risk (OR = 0.6, 95% CI = 0.4–0.9), and specifically use of aspirin. Further, the association with NSAID use was largely among invasive, high grade and TP53 positive tumors.

**Conclusion:**

While these agents have been investigated in several studies, a number of questions remain regarding the effects of analgesic and NSAID use on risk of bladder cancer.

## Background

Bladder cancer is the 4^th ^most common cancer in men and the 10^th ^in women [1]. While the main risk factor for bladder cancer is tobacco smoking, other important risk factors include certain occupational exposures such as aromatic amines and PAHs (polyaromatic hydrocarbons), exposure to water chlorination by-products [2], conditions causing chronic inflammation of the urological epithelium (e.g., infestation by *Schistosoma haematobium*) [2], and use of the immunosuppressant cyclophosphamide [3].

There is limited, however intriguing evidence on the effects of other drugs on bladder cancer risk, particularly analgesics and anti-inflammatory drugs (See additional file 1). Phenacetin, (N-(4-ethoxyphenyl) acetamide), an analgesic drug, was classified by the International Agency for Research on Cancer (IARC) in 1987 as being probably carcinogenic to humans (Group 2A) and analgesic mixtures containing phenacetin as being carcinogenic (Group 1) [4]. Two epidemiologic studies of bladder cancer included in the IARC report indicated an excess risks of two- to over six-fold associated with use of phenacetin containing drugs [[5,6], additional file 1]. One additional study found a four-fold increased risk of bladder cancer among phenacetin abusers [[7], additional file 1]. However, these were relatively small studies, and were not able to demonstrate dose-related effects. While subsequent studies, based on larger samples, are less clear [8-11], most suggest an elevated risk (see additional file 1). Although phenacetin has been discontinued in the US since the 1980s, whether users of phenacetin remain at increased risk of bladder cancer is not known as the latency period is potentially longer than two decades [12]. Further, phenacetin's metabolite paracetamol (also known as acetaminophen) also is related to an enhanced risk of renal cancer [9,11,13-19]. While some studies of bladder cancer found evidence of an elevated risk associated with heavy use of paracetamol, the majority did not, and some suggested an overall decreased risk [[6,8,10,11,13-15,19], additional file 1]. Studies to date on NSAIDs use and bladder cancer risk are mixed [8,20-25]. The largest of these found evidence of a reduction in bladder cancer risk (OR = 0.63) with greater than 1242 grams of aspirin use and greater than or equal to 168 grams of acetic acids (OR = 0.65) [8], whereas a study, performed on a general practitioner database in the UK (n = 1041), found no relation between any type of NSAID use and risk of bladder cancer. As yet, there are limited data on the effect of analgesics and NSAIDs on the incidence of specific clinical, histopathologic or molecular types of bladder cancer. Thus, in light of the currently inconclusive evidence on the potential impact of analgesic and anti-inflammatory drugs on bladder cancer occurrence, we assessed lifetime use of these drugs in relation to bladder cancer incidence in a population-based case-control study. We further specifically evaluated associations with stage, histologic grade and TP53 overexpression of the tumor.

## Methods

### Study group

Through the New Hampshire State Department of Health and Human Services' rapid reporting Cancer Registry, we identified newly diagnosed cases of bladder cancer among New Hampshire residents, aged 25–74 years during the July 1, 1998 to December 31, 2001 period. For efficiency, we shared a control group with a study of non-melanoma skin cancer covering a diagnostic period of July 1, 1997 to March 30, 2000. Controls less than 65 years of age were selected using population lists obtained from the New Hampshire Department of Transportation. Controls 65 years of age and older were chosen from data files provided by the Centers for Medicare & Medicaid Services (CMS) of New Hampshire.

We conducted standardized in-person interviews with study participants to obtain information on demographic traits, use of tobacco (including frequency, duration and intensity of cigarette smoking), alcohol, and other exposures. We requested the original paraffin-embedded tumor specimen for histopathology re-review by the study pathologist who classified tumors according to WHO ISUP criteria. Of the 342 cases with evaluable pathology, 327 (96%) were deemed cancerous by the study pathologist. Due to the high concordance rates, we classified subjects based on the original pathologist's diagnosis; whereas tumor morphology, extent of disease, and grade were based on the standardized histopathology re-review. Immunohistochemical analysis of the tumors was performed for TP53 and scored for intensity and percent of tumor cells staining positively, as described previously [26]. We obtained informed consent from each participant and all procedures and used study materials approved by the Committee for the Protection of Human Subjects at Dartmouth College.

### Drug use assessment

Subjects were asked if they used a pain medication at least four times a week for one month or longer prior to the reference date (diagnosis date of the cases and a comparable date randomly assigned to the controls). Those who responded positively were asked the brand names of each medication they took, and for each medication, the age began and stopped, total duration of use and the condition for which the drug was prescribed. Those who responded that they did not use pain medications at the specified frequency were considered non-users.

Brand names were recoded into active ingredients based on a drug matrix that took into account variations in the composition of the drugs over time, and specifically addressed the exact years of withdrawal/substitution of phenacetin from the formulation. Past editions of the Physician Desk Reference ^® ^were used as the main source of information on drug composition to generate the matrix. Whenever necessary, a variety of other sources were used, such as direct contact with pharmaceutical companies, documents from regulatory agencies and others. Drug categories considered for the analysis were: phenacetin, paracetamol, any NSAID (including aspirin, ibuprofen, diclofenac and any other drug generally included in this category), aspirin, and ibuprofen.

### Statistical analysis

We computed ORs and their 95% confidence intervals for bladder cancer risk for the studied drugs using logistic regression with those who responded that they did not use the pain medications at all or with the specified frequency as the reference category. Risk estimates were adjusted by age in quintiles according to the age distribution among the controls: (<52, 52 to 61, 62 to 67, 68 to 71, and >71 years), sex and smoking status (never, former, current). We further assessed the possibility that education, as a marker of socioeconomic status, could act as a potential confounder but the inclusion of this variable did not appreciably influence our results and therefore was not included in our final models.

To distinguish the effects of the specific types of NSAIDs and analgesic drugs, we used two models. One, the "adjusted model", simultaneously included each of the drug categories (i.e., phenacetin, paracetamol, aspirin, ibuprofen and the rest of NSAIDs with or without aspirin and/or ibuprofen) and the other, the "exclusive use" model, included subjects that had only used one specific type of pain medication. Duration was classified according to tertiles of control distribution for each drug. Tests for trend were performed by including a single term for categorical exposure variables in logistic regression models using non-users as the reference category.

Further, we examined a model in which the drug use variables excluded the year prior to the reference date in order to assess the possibility of drug use consequent to undiagnosed tumor symptoms. In addition to evaluating all bladder cancers combined, we evaluated risks by extent of disease (noninvasive versus invasive), for noninvasive tumors, grade (low grade versus high grade), and TP53 intensity (<3 versus 3+). In the analysis of TP53 intensity, we assessed whether further adjustment by stage or grade affected the results. We did not find evidence of such effects, and thus, did not include stage or grade in the final models of TP53 status. The statistical package SAS v9.1 was used for all the analyses.

## Results

### Associations with medication use

Of the 472 potentially eligible cases we contacted, 398 (84%) took part in the study, and from 376 (94%) of these, we obtained history of pain medication use. Of the 694 potential controls we contacted, 526 (76%) took part in the study, and from 463 (88%) of these, we obtained history of pain medication use. Subjects were mainly Caucasians and more than half were men (Table 1). More cases than controls were over age 60 years, former or current smokers, and did not have education beyond high school (Table 1). Overall, 42% of cases and 45% of controls reported regular use of a pain medication. Among controls, 8% were regular users of phenacetin (4% of women and 10% of men), 13% of paracetamol (18% of women and 9% of men), 27% of aspirin (20% of women and 32% of men), and 13% of ibuprofen (15% of women and 11% of men). Of the histologically reviewed cancers, 66% percent were superficial and noninvasive, 6% were *in situ *carcinomas, and 28% were invasive.

**Table 1:** Selected characteristics of bladder cancer cases andcontrols from New Hampshire

**Selected Characteristics**	**Cases**	**Controls**
*Total Subjects N (%)*	398 (100)	526 (100)
*Sex*		
Women	107 (27)	220 (42)
Men	291 (73)	306 (58)
*Race*^a^		
White	379 (98)	513 (99)
Other	8 (2)	5 (1)
*Age (Years)*		
≤ 51	57 (14)	106 (20)
52–61	108 (27)	123 (23)
62–67	90 (23)	112 (21)
68–71	89 (22)	103 (20)
>71	54 (14)	82 (16)
*Highest Level of Educational Attainment*^a^		
≤ High school	204 (53)	239 (46)
> High school	182 (47)	281 (54)
*Cigarette Smoking*^a^		
Never	66 (17)	193 (37)
Former	195 (50)	243 (47)
Current	129 (33)	85 (16)
*Amount Smoked (cigarettes/day)*		
≤ 12	41 (13)	90 (31)
12–20	132 (43)	119 (41)
>20	136 (44)	84 (29)
*WHO-ISUP classification*^b^		
Carcinoma *in situ*	18 (6)	-
Papilloma	1 (0)	
Papillary neoplasm of low malignant potential	84 (26)	-
Papillary carcinoma, low grade	112 (34)	-
Papillary carcinoma, high grade	74 (23)	-
Other	38 (13)	-
*Tumour grade*^*b*,*c*^		
Grade 1	145 (47)	-
Grade 2	61 (20)	-
Grade 3	103 (33)	-
*Tumour stage*^b^		
Superficial	216 (66)	-
Invasive	93 (28)	-
In situ	18 (6)	-

^a ^8 controls and 11 cases had no race information, 6 controls and 12 cases had no education information, 5 controls and 8 cases had no smoking information

^b ^Restricted to individuals with confirmed cancers on histopathology reviewed (n = 327).

^c ^Grade determinations exclude in situ lesions (n = 18).

Bladder cancer cases reported regular phenacetin use more frequently than control subjects (Table 2; OR = 2.2, 95% CI = 1.3–3.8). The odds ratio associated with phenacetin use was highest among those with longer duration of use (OR_>8 yrs _= 3.0, 95% CI = 1.4–6.5), although even short term users (4 years or less) had an increased risk (OR_≤4 yrs _= 2.2, 95% CI = 1.0–4.7) (P for trend = 0.005). We were unable to fit the exclusive use model for phenacetin because there were no exclusive users. In contrast, regular paracetamol use was not related to any statistically significant association with bladder cancer in the adjusted (OR = 0.7, 95% CI = 0.4–1.2) or exclusive use (OR = 0.8, 95% CI = 0.5–1.6) models (Table 2).

**Table 2:** Odds ratios (95% confidence intervals) for bladder cancer among regular users of phenacetin and paracetamol, with adjustment for other drugs, and among exclusive users.

**Drug use**	**Adjusted Model**^a^			**Exclusive Use Model**^b^		
	**Controls N(%)**	**Cases N(%)**	**OR (95%CI)**^c^	**Controls N(%)**	**Cases N(%)**	**OR (95%CI)**^c^
*Phenacetin*						
Never	421 (92.3)	313 (85.5)	1.0 (reference)			
Ever	35 (7.7)	53 (14.5)	2.2 (1.3–3.8)			
Duration of use^d^						
≤ 4 yrs	14 (3.1)	22 (6.0)	2.2 (1.0–4.7)			
4–8 yrs	9 (2.0)	6 (1.6)	1.1 (0.4–3.5)			
>8 yrs	12 (2.6)	25 (6.8)	3.0 (1.4–6.5)			
P for linear trend^e^			0.005			
*Paracetamol*						
Never	398 (87.3)	335 (91.5)	1.0 (reference)	249 (89.6)	211 (90.9)	1.0 (reference)
Ever	58 (12.7)	31 (8.5)	0.7 (0.4–1.2)	29 (10.4)	21 (9.1)	0.8 (0.5–1.6)
Duration of use^d^						
≤ 4.5 yrs	19 (4.2)	12 (3.3)	0.9 (0.4–1.9)	12 (4.3)	8 (3.4)	0.8 (0.3–2.0)
4.5–16 yrs	19 (4.2)	13 (3.6)	0.9 (0.4–1.9)	10 (3.6)	9 (3.9)	0.9 (0.4–2.5)
>16 yrs	20 (4.4)	6 (1.6)	0.4 (0.2–1.2)	7 (2.5)	4 (1.7)	0.8 (0.2–3.1)
P for linear trend^e^			0.116			0.672

^a ^Model adjusted by age, sex, number of cigarettes smoked per day, and use of other NSAIDs and analgesics

^b ^Model adjusted by age, sex, and number of cigarettes smoked per day

^c ^Excluded 3 subjects missing number of cigarettes smoked per day.

^d ^Based on tertile of duration of use among controls. No medication use is referent.

^e ^Tests for trend were performed by including a single term for categorical duration of medication use using non-users as the reference category.

NSAID use was associated with a reduced risk of bladder cancer (Table 3; adjusted model OR = 0.6, 95%CI = 0.4–0.9 and exclusive use model OR = 0.6, 95%CI = 0.4–0.9). The association with NSAIDs overall was largely due to aspirin (adjusted model OR = 0.6, 95%CI = 0.4–0.9 and exclusive model OR = 0.5, 95%CI = 0.3–0.8) (Table 3). While the crude prevalence rate of aspirin use was higher among cases than controls, a higher proportion of aspirin users also consumed phenacetin. Thus, lack of adjustment for phenacetin resulted in an odds ratio closer to unity (OR = 0.9; 95% CI = 0.7–1.3).

**Table 3:** Odds ratios (95% confidence intervals) for bladder cancer among regular users of NSAIDs, with adjustment for other drugs, and among exclusive users.

**Drug use**	**Adjusted model**^a^			**Exclusive use model**^b^		
	**Controls N(%)**	**Cases N(%)**	**OR (95%CI)**^c^	**Controls N(%)**	**Cases N(%)**	**OR (95%CI)**^c^
*All NSAIDs*						
Never	278 (61.0)	232 (63.4)	1.0 (reference)	249 (68.0)	211 (74.6)	1.0 (reference)
Ever	178 (39.0)	134 (36.6)	0.6 (0.4–0.9)	117 (32.0)	72 (25.4)	0.6 (0.4–0.9)
Duration of use^d^						
≤ 4 yrs	61 (13.4)	47 (12.8)	0.7 (0.4–1.1)	46 (12.6)	25 (8.8)	0.6 (0.4–1.1)
4–10 yrs	58 (12.7)	37 (10.1)	0.5 (0.3–0.8)	35 (9.6)	23 (8.1)	0.6 (0.3–1.1)
>10 yrs	59 (12.9)	50 (13.7)	0.7 (0.4–1.1)	36 (9.8)	24 (8.5)	0.6 (0.4–1.1)
P for linear trend^e^			0.020			0.029
*Aspirin*						
Never	333 (73.0)	263 (71.9)	1.0 (reference)	249(78.3)	211(84.4)	1.0 (reference)
Ever	123 (27.0)	103 (28.1)	0.6 (0.4–0.9)	69(21.7)	39(15.6)	0.5 (0.3–0.8)
Duration of use^d^						
≤ 4 yrs	43 (9.4)	32 (8.7)	0.5 (0.3–0.9)	27(8.5)	10(4.0)	0.4 (0.2–0.8)
4–9 yrs	41 (9.0)	24 (6.6)	0.3 (0.2–0.6)	21(6.6)	12(4.8)	0.5 (0.2–1.0)
>9 yrs	39 (8.6)	47 (12.8)	0.9 (0.5–1.5)	21(6.6)	17(6.8)	0.7 (0.4–1.4)
P for linear trend^e^			0.114			0.038
*Ibuprofen*						
Never	399 (87.5)	332 (90.7)	1.0 (reference)	257 (88.3)	214 (89.5)	1.0 (reference)
Ever	57 (12.5)	34 (9.3)	0.8 (0.5–1.2)	34 (11.7)	25 (10.5)	0.9 (0.5–1.6)
Duration of use^d^						
≤ 3 yrs	22 (4.8)	15 (4.1)	0.9 (0.5–1.9)	11 (3.8)	11 (4.6)	1.5 (0.6–3.7)
3–10 yrs	16 (3.5)	8 (2.2)	0.5 (0.2–1.3)	10 (3.4)	7 (2.9)	0.7 (0.3–2.0)
>10 yrs	19 (4.2)	11 (3.0)	0.8 (0.4–1.8)	13 (4.5)	7 (2.9)	0.7 (0.3–1.8)
P for linear trend^e^			0.244			0.484

^a ^Model adjusted by age, sex, number of cigarettes smoked per day, and use of other NSAIDs and analgesics

^b ^Model adjusted by age, sex, and number of cigarettes smoked per day

^c ^Excluded 3 subjects missing number of cigarettes smoked per day.

^d ^Based on tertile of duration of use among controls. No NSAID use is referent.

^e ^Tests for trend were performed by including a single term for categorical duration of NSAID use using non-users as the reference category.

Regular ibuprofen use was unrelated to risk of bladder cancer in the adjusted model (OR = 0.8, 95%CI = 0.5–1.2) and exclusive use model (OR = 0.9, 95%CI = 0.5–1.6). Exclusion of drugs used in the year prior to the reference date did not materially alter the risk estimates but compromised the statistical power of the analysis (data not shown).

We indirectly assessed the effect of dose of aspirin by using the condition for which aspirin was prescribed as a proxy. In general, aspirin is prescribed for coronary heart disease prevention at lower doses (i.e., ≤ 150 mg/day) than for inflammatory or painful conditions (i.e., ≥ 325 mg bid or tid). When we stratified by condition we did not find any differences in the risk estimates i.e., among aspirin exclusive users the odds ratio was 0.5 (95%CI = 0.3–0.9) for cardiovascular disease and 0.5 (95%CI = 0.2–1.3) for other conditions.

### Stratification by cell type, stage and grade

When we restricted the analysis of the effect of drugs to transitional cell carcinomas, excluding squamous cell carcinomas (n = 2), small cell carcinoma (n = 2), adenocarcinomas (n = 1), and others (n = 13), results remained unchanged (data not shown). Exclusion of papillary urothelial neoplasia of low malignant potential (n = 84), also yielded similar results for all drug use categories except everusers of phenacetin had a slightly higher bladder cancer risk (OR = 2.6, 95%CI = 1.5–4.6) without these cases.

In the analysis stratified by tumor phenotype, we found a stronger  inverse association with NSAID use for invasive, high grade non-invasive and TP53 positive tumors (Table 4). There were no consistent patterns by stage, grade or TP53 status with the other drugs.

**Table 4:** Odds ratios (95% confidence intervals) for bladder cancer among regular users of NSAIDs, with adjustment for other drugs – stratified by tumor invasion, tumor grade and TP53 IHC intensity.

**Drug use**^a^	**Invasion**		**Noninvasive**		**TP53 IHC Intensity**	
	**Noninvasive**	**Invasive**	**Low grade**	**High grade**	**<3**	**3+**
	**OR (95%CI)**^b^	**OR (95%CI)**^b^	**OR (95%CI)**^b^	**OR (95%CI)**^b^	**OR (95%CI)**^b^	**OR (95%CI)**^b^
*Phenacetin*	2.4 (1.3–4.5)	2.2 (0.9–5.1)	2.2 (1.1–4.2)	4.3 (1.0–17.9)	2.0 (1.1–3.8)	2.7 (1.2–6.5)
*Paracetamol*	0.6 (0.3–1.1)	1.1 (0.5–2.2)	0.5 (0.2–0.9)	2.0 (0.6–6.4)	0.4 (0.2–0.8)	1.7 (0.8–3.5)
*All NSAIDs*	0.7 (0.4–1.0)	0.5 (0.3–1.0)	0.7 (0.5–1.1)	0.4 (0.1–1.4)	0.8 (0.5–1.2)	0.5 (0.3–0.9)
*Aspirin*	0.6 (0.3–1.0)	0.4 (0.2–0.9)	0.6 (0.4–1.0)	0.5 (0.1–1.8)	0.7 (0.4–1.1)	0.4 (0.2–0.9)
*Ibuprofen*	0.7 (0.4–1.3)	0.7 (0.3–1.6)	0.8 (0.4–1.4)	0.3 (0.0–2.3)	0.9 (0.5–1.6)	0.6 (0.2–1.4)

^a ^Restricted to the 327 subjects having pathologic examination of tumor material and confirmation of diagnosis.

^b ^Referent category is never use of NSAID medications. Model adjusted by age, sex, number of cigarettes smoked per day, and use of other NSAIDs and analgesics.

## Discussion

Our findings suggest that regular use of NSAIDs, in particular aspirin, related to a lower risk of this malignancy. The association appeared strongest for invasive, high grade noninvasive and TP53 positive tumors. Also, our findings provide further evidence of an increased risk of bladder cancer among users of phenacetin-containing drugs, and additionally, suggest that risk is greatest among long term users.

Case-control studies like ours have the potential for recall bias. To enhance recall we showed lists of commonly used anti-inflammatory and analgesic medications. One concern is that the prevalence of recalled phenacetin use may be lower for recent studies because the drug was withdrawn from the market nearly two decades ago and recall of past NSAID use decreases with time since last use [27]. Arguing against the presence of recall bias is that the prevalence rates of analgesic and anti-inflammatory drug use in our study were similar to those of other studies conducted in the United States, Northern Europe and Australia [5,8,11]. If subjects had been aware of the relation between phenacetin and urothelial cancers, recall bias could, at least, partly explain the increased risk seen for this drug. It is worth noting, however, that phenacetin was withdrawn from the market because of its association with interstitial nephritis, not because of an increased risk of cancer. Thus, bladder cancer patients would probably not relate their cancer to their use of phenacetin in the past. Finally, people tend to remember the brands of the phenacetin-containing anti-flu medications (e.g., Repan, Norgesic, Phenaphen, Fiorinal and Darvon compound), more than the actual ingredients because these anti-flu preparations usually included a combination of analgesics (i.e., phenacetin antihistamines, decongestants, and other ingredients). Nevertheless, we cannot rule out the possibility that some degree of recall bias might have affected our results. For other analgesics and NSAIDs, we would not anticipate differential recall (i.e., between cases and controls) because these drugs are not generally perceived as modifiers of bladder cancer risk. Moreover, the effects of the various drugs were not all in one direction as would be expected in the presence of recall bias.

While we do not suspect differential misclassification (i.e., due to recall bias), non-differential misclassification likely exists in our data. We attempted to minimize this by defining drug use as frequent use (e.g., four times per week during a month or more). By doing this, the "non-users" category included subjects who could have used the drugs but did not fulfill our user definition. Such misclassification likely would lead to an underestimate of the effects.

We further need to consider the possibility of selection bias, i.e, that non-participants or those on whom we lacked drug use information differed from participants in ways that were not accounted for in the analysis. Overall participants and non-participants were generally similar according to factors we were able to evaluate: age, sex and urban residence (data not shown). Lack of adjustment for occupational history, exposure to water chlorination byproducts or other factors could potentially have biased our results. However, high risk occupational exposures or water chlorination exposure are unlikely to be related to the use of analgesics or NSAIDs, and adjustment for these factors did not affect the relation between medication use and risk of bladder cancer in a case-control study from Spain [28]. In our study, adjustment for educational level, did not influence our results. Moreover, if non-participants were more or less likely to use analgesics or NSAIDs, the resultant bias would be predicted to occur in a similar direction for all drugs (as with recall bias), which did not appear to be the case in our data. Perhaps a more important limitation of our analysis is lack of statistical precision, leaving chance as a possible explanation for our results.

Knowledge of the effects of phenacetin on urinary cancers largely derives from animal experiments and from studies of renal cancers for which a causal association has been established [4]. Relatively few epidemiologic studies have examined phenacetin and bladder cancer with published risk estimates ranging from 0.7 to 6.5 [[5,6,8,10,11], additional file 1]. Of the five prior studies conducted, only one observed no increase in bladder cancer risk among phenacetin users [11]. Our data further support an etiologic role of phenacetin in bladder cancer occurrence and they further suggest that risk increases with duration of use.

Paracetamol is a metabolite of phenacetin, but it is unclear whether paracetamol retains the carcinogenic potential of its parent compound. In our study, paracetamol was unrelated to bladder cancer risk. This agrees with the published literature which is generally consistent with no effect [6,8,10,11,13-15,19]. Paracetamol is not a potent inhibitor of cyclooxygenase (COX), but may inhibit NFkB, a transcription factor related to the inhibition of apoptosis [29], up-regulated in several cancers, including bladder cancer [30]. Metabolism of paracetamol results in a reactive metabolite (N-acetyl-P-benzoquinone imine (NAPQI)) that can form DNA adducts [31] and cause liver and renal toxicity [32]. Thus, paracetamol, in theory, could promote apoptosis through NFkB inhibition conferring protection against bladder cancer, or conversely, could act as a bladder carcinogen through accumulation of DNA adducts from its toxic metabolite NAPQI. Recent evidence also raises the possibility of a role of genetic variation of paracetamol metabolizing genes on bladder cancer susceptibility associated with paracetamol use [28]. Further investigation of genetic variation in the metabolic pathway of paracetamol and tumor phenotype in this and other populations may help to clarify the anti-carcinogenic or carcinogenic potential of paracetamol.

Aspirin and other NSAIDs are COX inhibitors (with varying isoenzyme affinities) and probably have alternative targets of action (i.e., NF kappa B inhibition) that could influence cancer occurrence [33]. COX-2 enzyme is over-expressed in most bladder tumors [34], and it is the inducible isoenzyme thought to promote carcinogenesis via induction of anti-apoptotic, proangiogenic and other tumorigenic stimuli and pathways involving the tumor suppressor gene TP53 [35]. COX-2 expression has been related to advanced bladder cancers, i.e., advanced stage and high grade histology [36]. These findings are consistent with the stronger association we observed with NSAID use among those with more aggressive tumors and those with TP53 alteration, suggesting that COX inhibition could be especially relevant for these poorer prognosis tumors. However, to our knowledge, no previously published studies have evaluated this.

Among the commonly prescribed NSAIDs, aspirin and ibuprofen are non-selective COX inhibitors, and diclofenac is COX-2 selective [33]. Randomized clinical trials assessing various NSAIDs for bladder cancer are currently underway, although results are still unavailable. Low dose aspirin (100 mg every other day) for an average of ten years did not lower bladder cancer incidence in the Women's Health Study randomized controlled trial (with 27 cases in the treatment and 24 cases in the placebo group) [37]. Our results generally are in agreement with a large population-based study from southern California [8] that observed a reduced bladder cancer risk particularly among heavy users of aspirin (OR = 0.63, 95%CI = 0.43–0.92 for the highest tertile of cumulative dose). However, other case-control studies have not reported a relation between the use of aspirin and the risk of bladder cancer [11,20]. Additionally, all prospective studies have been null although based on a limited number of bladder cancer events [21,23-25,38]. The most recent prospective cohort study [23] found an increased mortality from bladder cancer in women that had been users of aspirin (RR = 12.31, 95%CI = 2.98–50.80), based on 15 bladder cancer deaths, and no association in men. Use of bladder cancer mortality rather than incidence as the endpoint further complicates the interpretation of this and other studies as bladder cancer carries a relatively favorable prognosis. Thus, the potential role of aspirin on bladder cancer risk is as yet unresolved.

Regarding non-aspirin NSAIDs, the southern California study found a decreased bladder cancer risk among acetic acid NSAIDs users, with a suggestion of a dose-response effect (OR = 0.46, 95%CI = 0.21–1.03 for heaviest users). A record linkage study from Denmark with 330 bladder cancer cases, found that prescribed non-aspirin NSAIDs slightly increased the risk of bladder cancer (OR = 1.2, 95%CI = 1.0–1.3), although no dose-response relationship was observed [24]. Misclassification also is likely in studies relying solely on prescription data as many commonly used NSAIDs do not require a prescription. Further studies with a larger number of subjects may be able to discriminate the effects of specific NSAIDs more clearly.

## Conclusion

In conclusion, our data provide evidence that aspirin use may be related to a decreased bladder cancer risk, particularly for more advanced tumors and ones that contain TP53 alterations. They confirm previous findings on an increased risk of bladder cancer among users of phenacetin-containing medications, and additionally suggest that the risk relates to duration of use. Bladder cancer is the most common urologic malignancy in humans, and it typically carries a favorable prognosis, but mortality and morbidity, including a high risk of recurrence, remain important public health concerns. While several studies have investigated the role of NSAIDs and other analgesic medications, many issues remain. In light of the chemopreventive effects of NSAIDs including aspirin for other neoplasms, the possibility that they may reduce bladder cancer incidence warrants further consideration. Studies assessing the effect on bladder cancer prognosis are lacking but would be of great interest.

## Competing interests

The author(s) declare that they have no competing interests.

## Authors' contributions

JF carried out the variable creation, statistical analysis, and drafted the manuscript. MK participated in the drafting of the manuscript and in the analysis plan. MS assisted in the statistical analysis and variable creation. AS performed the histopathology reviews and assessment of p53 immunohistochemistry. AA assisted in the retrieval of pathology materials. JH provided urology expertise. KK leads the somatic alteration component of the project. MRK is principal investigator and participated in all phases of the study. All authors read and approved the final manuscript.

## Pre-publication history

The pre-publication history for this paper can be accessed here:



## Supplementary Material

Additional file 1 Literature review of the published evidence on the relation between analgesic and anti-inflammatory drug use and risk of bladder cancer. Click here for file
