# Supplementary material for: Analgesic and anti-inflammatory drug use and risk of bladder cancer: a population based case control study
Source: BMC Urol. 2007 Aug 10;7:13. doi: 10.1186/1471-2490-7-13 (PMC2018698; doi:10.1186/1471-2490-7-13)
Supplement: Additional file 1 — Literature review of the published evidence on the relation between analgesic and anti-inflammatory drug use and risk of bladder cancer. [file 1471-2490-7-13-S1.doc]

**Additional File 1- Literature review of the published evidence on the relation between analgesic and anti-inflammatory drug use and risk of bladder cancer**

| **First Author, Year** | **User Definition** | **Phenacetin** | **Paracetamol** | **Aspirin** | **Other NSAIDs** | **Comments** |
| --- | --- | --- | --- | --- | --- | --- |
| *Cohort Studies* |  |  |  |  |  |  |
| Friis S et al, 2002 [14] | Prescriptions during period 1989-1995 |  | OR = 1.1 (0.9-1.4)  Number of prescriptions:  1 prescription  OR = 1.1 (0.7-1.5)  2-4 prescriptions  OR = 1.6 (1.1-2.1)  5-9 prescriptions  OR = 0.8 (0.4-1.3)  ≥ 10 prescriptions  OR = 1.0 (0.7-1.5) |  |  |  |
| Sørensen HT et al, 2003 [24] | Prescriptions during period 1989-1995 |  |  |  | OR = 1.2 (1.0-1.3)  Number of prescriptions:  1 prescription  OR = 1.1 (0.9-1.3)  2-4 prescriptions  OR = 1.2 (1.0-1.4)  5-9 prescriptions  OR = 1.1 (0.8-1.4)  ≥ 10 prescriptions  OR = 1.2 (0.9-1.6) |  |
| Ratnasinghe LD et al, 2004 [23] | Recent use at time of inclusion in study (1976-1980). Follow up 12-16 years. |  |  | OR = 3.36 (1.03-10.97) |  | Adjusted for sex, BMI, race, poverty index, education, smoking, and age. |
| Kaye JA et al, 2001 (nested case-control) [15] | Prescriptions in years 1 to 5 before diagnosis |  | OR = 0.9 (0.7-1.3)  Number of prescriptions:  1-5 prescriptions:  OR = 0.8 (0.5-1.2)  6-19 prescriptions:  OR = 1.3 (0.8-2.2)  ≥ 20 prescriptions  OR = 0.7 (0.4-1.4) |  |  | Adjusted for sex, age, general practice, duration of prescription history in database, index date, smoking, and BMI. |
|  |  |  |  |  |  |  |
| *Case-Control Studies* |  |  |  |  |  |  |
| Fokkens W, 1979 [7] | > 2000g life-long | OR = 4.1, p = 0.02 |  |  |  | Adjusted for age and sex. |
| McCredie M et al, 1983 (women only) [5] | > 100g life-long | OR = 2.3 (1.4-3.9)  Cumulative Dose (g or kg):  100g-10kg:  OR = 2.3, p< 0.001  >10kg:  OR = 2.7, p< 0.001 |  |  |  | Adjusted for age and smoking |
| Piper JM et al, 1985 (women only) [6] | > 30 days/year life-long | OR = 6.5 (1.5-59.2) | OR = 1.5 (0.4-7.2) |  |  | Adjusted for race, educational level, smoking, history of bladder infections, and history of exposure to radioactive iodine. |
| McCredie M et al, 1988 [10] | >100g life-long | OR = 2.1 (1.3-3.5)  Cumulative dose (kg):  >1 kg:  OR = 2.0 (1.1-3.5) | OR = 0.7 (0.4-1.3)  Cumulative dose (kg):  >1 kg:  OR = 0.7 (0.4-1.3) |  |  | Adjusted for sex, smoking, and paracetamol use. |
| Derby LE et al, 1996 [13] | ≥ 1 prescription since 1976 |  | Number of prescriptions:  1 prescription  OR = 1.0 (0.8-1.4)  2-9 prescriptions  OR = 1.0 (0.8-1.3)  10-19 prescriptions  OR = 1.1 (0.6-2.0)  20-39 prescriptions  OR = 1.1 (0.6-2.3)  ≥ 40 prescriptions  OR = 1.3 (0.6-2.8) |  |  | Adjusted for smoking, BMI, history of urinary tract infections, occupation, and coffee drinking |
| Rosenberg L et al, 1998 [19] | ≥2 times per week & ≥1 month life-long |  | OR = 1.1 (0.6-1.9)  Duration of use (years):  <5 years of use:  OR = 1.1 (0.5-2.3)  ≥5 years of use:  OR = 1.1 (0.5-2.6) |  |  | Adjusted for age, sex, interview year, and geographic area. |
| Pommer W et al, 1999 [11] | >1 kg life-long | OR = 0.75 (0.39-1.43) | OR = 0.83 (0.33-2.07) | OR = 1.09 (0.73-1.64) |  | Adjusted for smoking, ex-smoking, socioeconomic status, and laxative intake. |
| Langman MJS et al, 2000 [20] | Prescriptions in months 13 to 36 before diagnosis |  |  | Number of prescriptions:  Aspirin plus other NSAIDs:  1 prescription:  OR = 1.38 (1.08-1.75)  2-6 prescriptions:  OR = 1.09 (1.04 -1.40)  ≥7 prescriptions:  OR = 1.14 (0.85-1.53) |  | Adjusted for age and smoking |
| Castelao JE et al, 2000 [8] | ≥2 times per week ≥ 1 month life-long | OR = 1.52 (0.85-2073)  Cumulative dose (g):  <46g:  OR = 1.35 (0.60-3.07)  46-250g:  OR = 1.64 (0.73-3.70)  >250g:  OR = 1.85 (0.77-4.43) | OR = 0.85 (0.60-1.19)  Cumulative dose (g):  <114g:  OR = 0.75 (0.47-1.20)  114-885g:  OR = 0.68 (0.43-1.09)  >885g:  OR = 1.43 (0.87-2.35) | OR = 0.88 (0.70-1.12)  Cumulative dose (g):  <245g:  OR = 0.99 (0.73-1.34)  245-1242g:  OR = 0.94 (0.068-1.30)  >1242g:  OR = 0.63 (0.43-0.92) | Acetic acids  OR = 0.54 (0.31-0.94)  Proprionic acids  OR = 0.70 (0.49-0.99)  Cumulative dose (g):  Acetic acids  <168g:  OR = 0.92 (0.43-1.97)  ≥168 g:  OR = 0.65 (0.31-1.37)  Proprionic acids  <144g:  OR = 0.81 (0.51-1.28)  ≥144g:  OR = 0.55 (0.34-0.87) | Adjusted for education, smoking, occupation, NSAID use, and analgesic use. |
